# The experiences of intensive care nurses coping with ethical conflict: a qualitative descriptive study

**DOI:** 10.1186/s12912-023-01612-2

**Published:** 2023-11-30

**Authors:** Yuanfei Liu, Liying Ying, Yuping Zhang, Jingfen Jin

**Affiliations:** 1 Department of Nursing, The Second Affiliated Hospital Zhejiang University School of Medicine (SAHZU), Zhejiang University, Hangzhou, China; 2 Department of Nursing, School of Medicine, Zhejiang University, Hangzhou, China; 3 Changxing Branch Hospital of SAHZU, No.66 Taihu middle road, Changxing Country, Huzhou, 313100 Zhejiang China; 4 Key Laboratory of The Diagnosis and Treatment of Severe Trauma and Burn of Zhejiang Province, Hangzhou, China

**Keywords:** Coping strategies, Critical care nursing, Ethical conflict, Qualitative research, Thematic analysis

## Abstract

**Background:**

The critical conditions and life risk scenarios make intensive care nurses susceptible to ethical conflict. Negative consequences were recognized at both the individual level and the professional level which highly compromised the patient care and nurses’ well-being. Therefore, ethical conflict has become a major concern in nursing practice. However, the experience of coping with ethical conflict among intensive care nurses remains unclear.

**Aims:**

This study aims to explore the experience of intensive care nurses coping with ethical conflict in China.

**Methods:**

From December 2021 to February 2022, in- depth interviews with 15 intensive care nurses from five intensive care units in a tertiary general hospital in China was performed using purposive sampling. An inductive thematic analysis approach was used to analyze the data. We applied the consolidated criteria for reporting qualitative research for this study.

**Results:**

Two distinctive themes were found: detachment and engagement, which contained four subthemes: ignoring ethical problems in the workplace, seeking ways to express emotions, perspective-taking, and identifying positive assets. Theses coping strategies demonstrated an ongoing process with different essential features.

**Conclusion:**

This study provides a new insight into the experience of intensive care nurses coping with ethical conflict in clinical nursing. Intensive care nurses demonstrated differential experience of coping with ethical conflict including problem-focused, emotion-focused and meaning-making strategies. These findings have implications for policymakers and nursing administrators to develop ethical education and training and supportive environment for intensive care nurses to tackle this issue.

**Supplementary Information:**

The online version contains supplementary material available at 10.1186/s12912-023-01612-2.

## Introduction

Nursing practice is laden with ethical issues. Nurses play a pivotal role in healthcare than any other types of healthcare professionals due to the longer time spent with patients and their families. Providing appropriate nursing care seems to be a physically demanding and intellectually challenging process which involves various ethical problems and conflicts [1]. Due to the increasing demands of patients and their families, ethical conflict has become clearly evident and far more complicated in nursing practice [2].

Intensive care unit (ICU) is one of the most stressful clinical settings. Nurses working in ICU are at higher risk of confronting physical restraints, futile analgesia, palliative care, end-of-life decisions and numerous complex and unstable situations [3, 4]. The critical conditions and life-threatening scenarios make intensive care nurses susceptible to ethical conflict. To make matters worse, they often have low discretion in clinical decision-making and stay marginalized to express their voices due to the hierarchy in the workplace [5]. Hence, ethical conflict is pervasive in critical care setting. Examples include taking care of a patient who should in a general ward rather than an ICU, implementing an aggressive treatment that causes additional suffering in the patient, or making the best use of available techniques and resources for critically ill patients without significantly improvement in their outcomes [4, 6]. The phenomenon of ethical conflict demonstrated various adverse consequences at both the individual level and the professional level, such as compassion fatigue, professional burnout, job dissatisfaction and worsened nursing care [7–9]. The compromise of nurses’ well-being and patient care has highlighted the significance of ethical conflict in nursing practice.

In reviewing the literature, a few studies reported the coping strategies that nurses probably exert to tackle this problem. Nurses from psychiatric wards in Norway demonstrated avoidant and submissive patterns of behavior towards ethical conflict [10]. Similarly, Pavlish et al. [11] also found that healthcare providers in the US kept silent and shared a culture of avoidance when ethical conflict occurred. Moreover, being alone and uncertain and acting against conscience were found as the dominant ways of coping among nurses in Sweden in dialysis care setting [12]. By contrast, Kim et al. [13] interviewed 26 Korean nurses in a general hospital and identified the essential experience was being faithful to the nature of caring. Since ethical conflict has been found to be culture and context sensitive, the coping strategies demonstrate disparity in different culture and social contexts. Ethical principles that guide nurses to make ethical decisions may vary from culture to culture and nation to nation. In western countries, patients’ autonomy may be paid more attention and the medical discussions engage the patients directly [14]. However, in most traditional eastern countries, intensive care nurses often start the conversations with family members in truth-telling without the patients being fully informed [15]. Even though the Code of Ethics was established to provide formal statement and guidance for nurses both nationally and worldwide [16], such general guidance hardly provides evidence for nurses to cope with ethical conflict in nursing profession.

Although previous studies have paid attention to the coping behaviors of ethical conflict among nurses, little is known about this situation in China. Chinese nurses, like many nurses from other countries, often encounter some challenges that affect their ethics and values in practice. Given the nature of ICU setting and the devastating consequences of ethical conflict among intensive care nurses, identifying their experience of coping with ethical conflict would provide fundamental basis for strategy development. These experiences are subjective and influenced by various factors, such as personal values, professional codes, organizational policies, and cultural norms. As quantitative study is eclipsed in exploring contextual depth and understanding complex phenomenon, qualitative research can help capture the nuances and meanings of these experiences and provide insights into how the nurses cope with ethical conflict and what challenges they may encounter. Therefore, we conducted a qualitative descriptive study among intensive care nurses in China to identify their coping experience of ethical conflict and explore the potential problems and dilemma, which would contribute to the development of supportive intervention for intensive care nurses.

## Methods

### Design and setting

We performed a qualitative descriptive study on the experience of intensive care nurses coping with ethical conflict, using individual semi-structured in-depth interviews. This study was carried out from December 2021 to February 2022 in a public tertiary hospital in Hangzhou, China which contains six ICUs with over 300 intensive care nurses.

### Participants

Participants were recruited using purposive sampling. The inclusion criteria were intensive care nurses who were registered in China and had more than one year experience in critical care. Nurses who were not on duty or had an intern in ICU during the interview were excluded. Maximum variation sampling [17] was used regarding gender, age, professional title, department and years of engagement in clinical nursing. Since the main researcher (Y.L) was a graduate student in this hospital at the time, it was not hard to reach out the participants. The head nurses of each ICU helped select the participants who met the eligible criteria. After reaching out and identifying participants, we explained the anonymity, confidentiality and the information about the study to the participants before obtaining their consent. Only the researchers had access to the interview data. Participants were given the chance to terminate their involvement in the study at any time.

### Data collection

One main researcher (Y.L) conducted the face-to-face, semi-structured in-depth interviews at a quiet and private office or duty room in the hospital which was chosen by the participants and at pre-arranged time. Based on the literature review and the methodology of Kallio et al. [18], we developed the initial interview guide. A meeting with the research team was held to evaluate the interview guide by removing ambiguous or leading questions. Next, we invited an ethics specialist to assess whether it was comprehensive for the purposes of this study and whether the wording is appropriate. The interview guide was refined into several broad questions and some revisions of wording were made. The final version is presented in Table 1. After that, the interview guide was piloted with two participants. Follow-up questions and probing questions were used according to the situations to encourage elaboration of responses and ensure clarity when necessary. Since there were no further changes, the pilot data were included in the study.

**Table 1:** Interview guide

Number	Questions
1	Please describe events in your practice that you perceived as ethical conflict.
2	Were there anything you did to help yourself feel better or to get through?
3	Why you chose these ways to cope with these events?
4	Have your coping strategies changed a lot?
5	What were the factors that affected your coping strategies?
6	Do you have anything else to add or share?

The interviews language was Chinese and the data was generated by the main researcher (Y.L). The interviews were audio-recorded and lasted 50 to 73 min. Field notes about non-verbal information were also recorded during interviews. Since the researcher was the research instrument, the impact of subjectivity on the findings was considered. To avoid that, a reflexive journal was kept to record the researcher’s thoughts, problems and any other ideas during the study process. Data collection occurred concurrently with data analysis. Sample size was continuously monitored to identify when thematic saturation was reached. After 15 interviews, study themes were tentatively established. We then interviewed one additional intensive care nurses and did not notice new themes. Hence, the data collection continued until no new emergent themes.

### Data analysis

The audio recordings of the interview were transcribed verbatim and submitted to Nvivo 12 (QSR International) for analysis. Before that, transcripts were returned to participants for comment and correction. We removed the names of the transcripts and used numbers to assure confidentiality. An inductive thematic analysis approach (a bottom-up method whereby themes were generated from data, not pre-existing theory) was used as it can help identify both manifest and latent aspects and form in-depth understandings of a phenomenon [19–21]. We followed a six-step framework to draw the themes [19]. *(1) Being fully familiar with the data*: two researchers (Y.L and Y.Z), who were graduate nursing students with rich experience in qualitative research, independently read and reread the transcripts and took notes for formulating hidden codes using semantic coding approach. (*2) Creating initial codes*: a list of codes were generated and modified during the open coding process to identify interesting and noteworthy features in the transcripts and label paragraphs that contained relevant information. *(3) Searching for themes*: various themes were formed by identifying consistent patterns among codes. *(4) Reviewing themes*: the two researchers jointly reviewed the themes they had extracted independently to reach a shared agreement by combining, splitting, creating or discarding themes. *(5) Defining and name themes*: meeting with the rest of the authors was held regularly to resolve discrepancies and revise themes iteratively. *(6) Writing the report.* During the process, coding and theme generation were conducted in Chinese and only translated into English with semantic equivalence when writing the report. The researchers also wrote memos to record their thoughts and questions for discussion.

### Rigor

Lincoln and Guba’s criteria for rigor [22] (credibility, transferability, confirmability and dependability) was used. To ensure credibility, the researchers had at least six months of clinical experience in ICU and were trained in qualitative study, one of whom (J.J) was an expert in critical care. For transferability, the method of maximum variation sampling guaranteed the representation of the participants. Confirmability was addressed by using quotes from the participants to explicate each theme and verifying the results by the remaining authors. To enhance Dependability, interview guide was reviewed and revised by an ethics expert and research team prior to data collection. During the interviews, the questions were asked in a neutral and non-judgmental way. Data collection occurred concurrently with data analysis to ensure thematic saturation. We applied the consolidated criteria for reporting qualitative research (COREQ) [38] for this study (Supplementary Table 1).

## Results

**Fig. 1:**
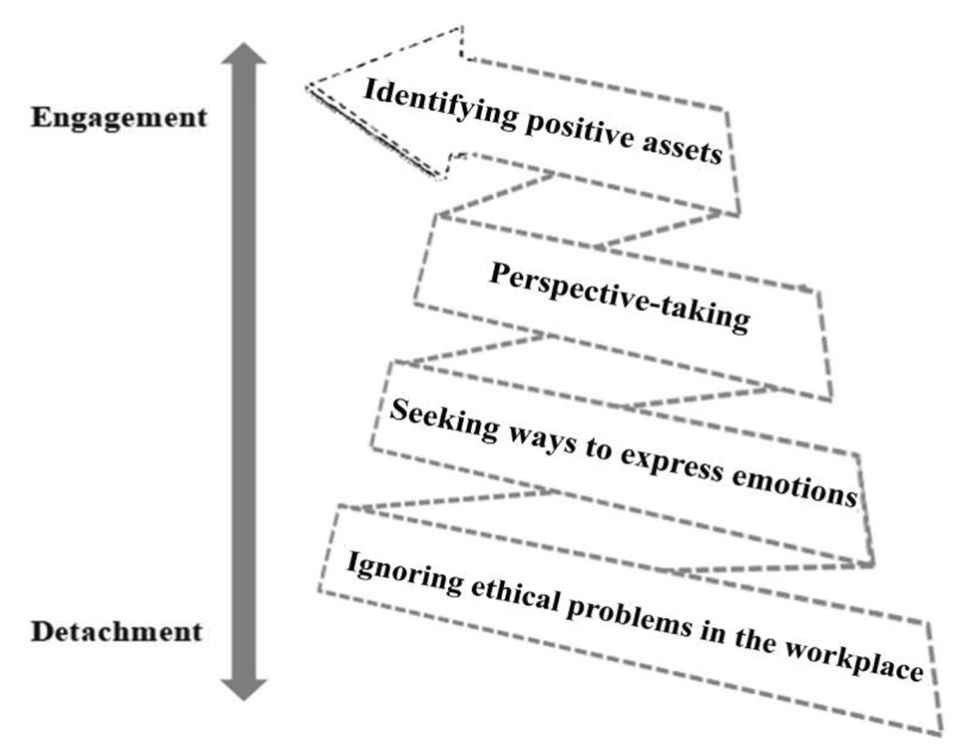
Relationship between themes and subthemes

15 intensive care nurses took part in the study, with 9 female and 6 male. The mean work experience in ICU was 8.3 years (2 to 22 years). The majority were staff nurses, aged 25–35, had received bachelor degree, and had worked in ICU for 1–10 years. Their detailed characteristics are shown in Tables 2 and 3. In this study, two main themes are identified: detachment and engagement, which represented two distinctive types of coping strategies. The four subthemes were: ignoring ethical problems in the workplace, seeking ways to express emotions, perspective-taking, and identifying positive assets, which reflected a dynamic process from detachment to engagement (Fig. 1).

**Table 2:** General characteristics

Characteristics	No.(n = 15)
Gender	
Female	9
Male	6
Age (years)	
18–24	1
25–30	5
31–35	4
36–40	3
41–45	1
≥ 46	1
Highest academic qualification	
Bachelor degree	13
Master degree	2
Years of experience in ICU	
1–5	6
6–10	5
11–15	2
16–20	1
＞20	1
Position	
Director of nursing	2
Nurse supervisor	5
Staff nurse	8
ICU nursing specialist or not	
Yes	2
No	13
Types of ICU	
Emergency	4
Surgical	2
Cardiovascular	3
Neurosurgical	4
General	2
Duties other than patient care	
Administrative	2
Educational	5
Neither	8

**Table 3:** Participant detailed information

No.	Gender	Highest academic qualification	Position	Experience in ICU (year)	Types of ICU	Other duties
1	F	Bachelor	Nurse supervisor	8	CSICU	Educational
2	M	Bachelor	Staff nurse	4	CSICU	Neither
3	F	Bachelor	Director of nursing	18	NICU	Administrative
4	M	Bachelor	Nurse supervisor	12	EICU	Educational
5	F	Master	Staff nurse	4	EICU	Neither
6	F	Bachelor	Director of nursing	22	SICU	Administrative
7	M	Bachelor	Staff nurse	5	CSICU	Neither
8	F	Bachelor	Staff nurse	2	EICU	Neither
9	F	Bachelor	Nurse supervisor	10	EICU	Educational
10	M	Bachelor	Staff nurse	4	SICU	Neither
11	M	Master	Staff nurse	8	NICU	Neither
12	F	Bachelor	Nurse supervisor	10	General	Educational
13	M	Bachelor	Staff nurse	2	General	Neither
14	F	Bachelor	Nurse supervisor	10	NICU	Educational
15	F	Bachelor	Staff nurse	6	NICU	Neither

EICU: Emergency Intensive Care Unit; SICU: Surgical Intensive Care Unit; CSICU: Cardiac Surgical Intensive Care Unit; NICU: Neurosurgery Intensive Care Unit

### Detachment

Detachment means that intensive care nurses look at the stressful situations in ethical conflict objectively, which seems to be a process of letting go. The essential characteristic is being disconnected from one’s feelings, comprising of ignoring personal values in the workplace and finding ways to express emotions.

### Ignoring ethical problems in the workplace

Intensive care nurses reported that ignoring ethical problems in the workplace was an effective way to help them avoid being overly emotionally affected by ethical conflict. It would be normal to come across various clinical situations that bring about ethical problems. Ideally, maintaining fairness of resource distribution and optimizing the benefits of patients who are in need have always been the cornerstone of healthcare. However, workplace constraints, such as physician-dominated decision-making, shortage of nursing staff and limited time and resources, may prevent nurses from applying universal principles in the practice. These problems existing in the healthcare organization and system can hardly be addressed by the individual and in a short term, thus, probably making intensive care nurses feel impotent and powerless. Consequently, some of them tended to ignore the problems that would trigger ethical conflict in the workplace.

*“I feel compelled to carry out the procedures decided by the doctors such as inserting a urinary catheter, though the patients are averse to that. I don’t think I’m a patient advocate in this case. But I have to do as told and set this problem aside.”* stated by nurse-5 who has worked in ICU for 4 years.

*“Normally, I take care of 4–5 patients, all of whom may be critically ill, so I have to get the work done quickly without providing psychosocial care for the patients. Actually, I don’t have time to consider this.”* expressed by nurse-10 who has worked in ICU for 4 years.

Another aspect of ethical problems was related to socioeconomic disadvantage. Many important forces shaping access to care and quality of care have more to do with social and economic status than with any particular medical treatments or procedures. The long stay in the ICU could bring about a colossal expenditure of time and finance, which added the burden on the entire family. Particularly for the low-income family, they may fall into impoverishment and indebtedness because of illnesses. Intensive care nurses were possibly trapped in the dilemma between patients’ poor outcomes and their financial burden. Furthermore, scarce resources were likely to exacerbate ethical problems as fairness of healthcare distribution might be compromised. These tricky situations went beyond the capacity and responsibility of intensive care nurses. They would rather detach themselves from the ethical issues which may otherwise rob their well-being and create vulnerability.

*“I witnessed the family took the patient home due to the financial catastrophe. I am wondering whether healthcare is a right or a privilege. But I can’t change that. I just let these problems go.”* noted by nurse-13 who has worked in ICU for 2 years.

“*The patient beds are always limited, but some of the VIP patients are not the most critically ill ones. These situations are common and gradually I just put them away.”* indicated by nurse-7 who has worked in ICU for 5 years.

*“I simply think about what I can and can’t do for patients, trying to look at ethical problems through the lens of an objective onlooker. No need to feel torn between anything we are unable to change. I don’t know whether it’s a good sign or not, but it can help me avoid distress.”* said by nurse-6 who has worked in ICU for 22 years.

### Seeking ways to express emotions

Intensive care nurses expressed that seeking ways to express emotions helped them get out of the trap of ethical conflict. There were numerous struggling scenarios in critical care setting that could lead to ethical conflict, such as additional suffering in the end-of-life care, ineffective pain management, limiting life-sustaining procedures, etc. When encountering ethical conflict, intensive care nurses probably suffered from overwhelmingly negative emotions including frustration, anxiety, distress, pain and anguish. They were also likely to feel powerless and marginalized in such situations due to the lack of discretion in decision-making. Additionally, working with professionally incompetent medical staff was another frequent source of ethical conflict and brought about a sense of helplessness and anger among intensive care nurses. These negative feelings were specific psychological responses to ethical conflict and took a toll on nurses’ overall well-being.

Since excessive and persistent psychological stress may lead to adverse outcomes both physically and mentally, intensive care nurses explored personal outlets to release negative emotions of ethical conflict. They tended to exert sociable and non-sociable approaches to express their feelings outside of work and create happiness, which may lead them to a well-adjusted and purposeful life. For example, they not only joined activities with personal motivations such as aesthetic or athletic pursuits, but also interacted with family or social events to maintain connections.

*“When I see the bedridden patients having been intubated for months, the end-of-life patients receiving aggressive treatments rather than palliative care, I feel painful as well. I can’t help myself crying to release my struggling feelings.”* described by nurse-15 who has worked in ICU for 6 years.

*“Sometimes it appears meaningless to prolong an irreversible terminal process, but withdrawing life-sustaining measures is also cruel. I often turn to my friends for catharsis and let myself be vulnerable or authentic. If they can feel related to me, I will feel much better.”* stated by nurse-1 who has worked in ICU for 8 years.

*“When I was single, I used to head out to a Karaoke bar with my besties. But, after we all get married, hardly can we find the same time available. I often fall out with my husband. It serves as a kind of outburst of emotions right now.”* expressed by nurse-9 who has worked in ICU for 10 years.

*“Shopping is the best painkiller for me. I also join volunteer work and social clubs like reading or running for fun at times. I keep maintaining these critical social activities.”* indicated by nurse-12 who has worked in ICU for 10 years.

### Engagement

Engagement means that intensive care nurses feel mentally and emotionally connected to the nursing scenarios of ethical conflict, indicating positive thinking regarding ethical issues. The essential characteristic is developing a new outlook and resilience, comprising of perspective-taking and identifying positive assets.

### Perspective-taking

Perspective-taking was described as a powerful tool to resolve ethical conflict by intensive care nurses. Based on the feeling of resonance, it was the ability to step into others’ shoes and understand their situations, feelings and motives. It was not uncommon that patients and families had radically different views with health professionals on many aspects of healthcare including treatment and medication, which may lead to incompatible values and competing interests, thus ethical conflict. However, intensive care nurses possibly used their own life experience and professional knowledge to make meaning of patients’ and their family members’ thoughts and behaviors. The competency to identify and understand the diverse values can be attributed to several factors such as years of work, educational background and personal experience. As for newly-graduated nurses or staff nurses, they learned to imagine the possible reasons behind the perceptions of patients and family members, so as to have a vicarious experience.

*“Sometimes we have to use all available technical and medical resources at the request of the family and this cause great ethical conflict. But, they just hope to indicate Xiao (filial piety) which is deeply rooted in Chinese culture.”* said by nurse-2 who has worked in ICU for 4 years.

*“When I cannot do anything but limit life support procedures due to the economic hardship in the family, I try to shift perspective to understand them — It seems to be a kind of relief.”* noted by nurse-8 who has worked in ICU for 2 years.

On the other hand, senior intensive care nurses typically became more sensitive to ethical conflict due to the higher level of exposure compared with the younger counterparts. Yet they were able to be open-minded and better comprehend family caregivers’ decisions. Since they had more experience in caring-related communications, they were more skilled to explore the family’s problems and difficulties, and get their messages across effectively. It was challenging to take more responsibility for senior nurses in critical care practice, but their personal experience helped them feel related and identify understandable motivations from others’ perspectives.

*“To love is to suffer. The patient with terminal tumor was on an irreversible way to the end of life, but his family requested to keep providing healthcare. In fact, the patient served as a critical anchor for them to be meaningful in the world,”* expressed by nurse-14 who has worked in ICU for 12 years.

*“They gave up, but I know it’s impossible for a family to save one in a desperate attempt. Various factors need to be taken into consideration such as the cost of living and kid education. The alive still need to move on. Otherwise, the life quality of the entire family may be compromised,”* said by nurse-4 who has worked in ICU for 12 years.

*“Euthanasia is illegal in China, but DNR (do not resuscitate) is a useful tool to preserve family dignity when they suffer from financial burden. Rationalization of DNR is helpful to relieve my sense of ethical conflict,”* indicated by nurse-11 who has worked in ICU for 8 years.

### Identifying positive assets

It is true that intensive care nurses easily fall victim to ethical conflict which is embedded in healthcare and may linger for years. As mentioned by nurse-3, *“it’s hidden in my heart and can be recalled even after two decades when a mid-aged man who had been so strong became so vulnerable and painful and gave up curative treatment.”* However, intensive care nurses were able to identify the positive assets from the evils and develop reflection on their own life. For example, some of them were equipped with a questioning mind to dig up the answer to the ethical issues.

*“I like to ask questions and embrace new knowledge and skills either professional or interest-related, so I can be better prepared for the potential ethical conflict.”* described by nurse-5 who has worked in ICU for 4 years.

Also, nurses identified the merit that the nursing scenarios leading to ethical conflict often reminded them of their own parents and reinforced the responsibility that they should shoulder.

*“When I’m trapped in ethical conflict regarding patient options in end-of-life care, I deeply believe that I should spend more time with my parents. When they get older, let them pass away peacefully without any suffering.”* stated by nurse-10 who has worked in ICU for 4 years.

For middle-aged nurses, the scenarios triggering ethical conflict rendered them risk-averse. They probably purchased health insurance for the family and saved money to reduce the uncertainty. Even though ethical-related cases distressed intensive care nurses, their multiple roles such as breadwinner, child rearers and caretakers of the elderly made them mature and responsible. They would like to make a living will declaration as they imagined the days when getting older.

*“I’m not sure whether the family’s decision aligns with what the patient really wants. This possibly traps me in ethical conflict. But it also exerts many positive influences on my life. I’d like to take advanced actions, for example, to decide what kind of healthcare I want to receive if I’m incapacitated, so when the day comes, I won’t bring any burden on my family,”* noted by nurse-9 who has worked in ICU for 10 years.

As they didn’t have children living at home, their attention was paid back to themselves. Ethical conflict appeared to bring perks that made up for the negative experience. Intensive care nurses became mindful and constructed personal worldview. They exhibited self-reflection at work which in turn made it possible to have a clear conscience. A positive attitude towards aging was also developed. They gave the top priority to the family which was central in life.

*“Instead of feeling down, I’m very motivated and energetic at work. I always try my best to encourage the patients and family caregivers. No matter what result it will be, I won’t feel struggling,”* expressed by nurse-3 who has worked in ICU for 18 years.

*“A granny passed away when her children were living aboard and didn’t manage to come back due to the pandemic. This experience of ethical conflict reminds me of myself. The traditional view of family bonding is embedded in Chinese people. It’s necessary to cherish every moment with the family who makes who we are and appreciate the ordinary but great life.”* indicated by nurse-6 who has worked in ICU for 22 years.

Overall, intensive care nurses illustrated various experience of coping with ethical conflict. According to the degree of connection between the individuals and the events, two distinctive coping strategies were identified as detachment and engagement. There was also a continuum between the two states including ignoring ethical problems in the workplace, seeking ways to express emotions, perspective-taking, and identifying positive assets, with different essential characteristics. The coping strategies adopted by intensive care nurses may be changed during the nursing profession.

## Discussion

In this study, we explored the experience of intensive care nurses coping with ethical conflict in China. The core findings was that intensive care nurses demonstrated detachment and engagement to tackle ethical conflict in clinical scenarios. The two distinctive states can be outlined as four types of strategies: ignoring ethical problems in the workplace, seeking ways to express emotions, perspective-taking, and identifying the positive assets. Despite the fact that ethical conflict was embedded in critical care, the level of ethical conflict varied among nurses [23]. Therefore, our study deepens the professionals’ understanding of ethical conflict in ICU setting and provides evidence to develop effective coping strategies and institutional support to mitigate this issue in the critical care context.

The four types of coping experience between detachment and engagement of ethical conflict in this study have similarities to the conceptual model proposed by Falcó-Pegueroles, comprising a continuum between the absence and presence of ethical conflict [24]. While this model worked as a tool to explain the difficulty in making morally correct decisions, both of them took into account the negative and positive impacts of ethical conflict on intensive care nurses. The absence of ethical conflict was corresponded to the detachment strategy which did not involve the emotion and stance on a matter of ethical concern. Likewise, the presence state aligned with the engagement strategy which highlighted the interaction between individuals and external events. Based on the two ends of the model, we elaborated four coping strategies to help understand the multi-faceted ethical conflict in critical care nursing.

One of the detachment strategies was to ignore ethical problems in the workplace. It is true that nursing profession features various principles and values which act as guide in providing healthcare [25]. Nurses especially the newly graduated may believe what they have learned to be true and act accordingly [26]. However, there was often a gap between the ethical codes and the application in practice [27, 28]. Intensive care nurses need to identify and respond to the potential ethical problems. When nurses came across the conditions that went beyond their control and capacity such as resource distribution and health inequity, they tended to ignore the ethical problems in the workplace. This was consistent with the findings in an acute psychiatric setting where nurses loyally did as told and tried to become immune to ethical conflict [10]. The problem-focused coping strategy was regarded as a process of letting go. On the one hand, it would help individuals look at the conflicting issues objectively with greater clarity [29], but on the other hand, it revealed the powerlessness of nurses in the face of ethical conflict. The nexus of socioeconomic issues, public health issues, law and policy possibly serve as the antecedents of ethical conflict. Hence, ethical conflict seems to be an institutional problem experienced at a personal level. The roots of ethical conflict should be addressed by the collaboration of academic educators, policymakers, institutional stakeholders and healthcare administrators.

Seeking ways to express emotions was described as another strategy of detachment. In fact, the nature of ethical conflict is an emotional response [30]. Nurses were more susceptible to ethical conflict compared with other health providers due to the longer stay with patients and female majority [31]. When facing ethical conflict, intensive care nurses probably suffered from various emotions and feelings such as moral indifference, moral uncertainty, moral distress and moral outrage in different nursing scenarios [24]. These troubling feelings were instinctive reactions to ethical conflict and might compromise nurses’ mental well-being. The emotion-focused coping strategies demonstrated their demand for social support and directly distracted them from ethical conflict. Similar to work-related stress and burnout, there were numerous interventions such as emotion management training and mindfulness that helped nurses release negative feelings and improve mental health [32]. However, to tackle ethical conflict needs more than a simple delivery of support services. The favorable practice environments should be built to enhance the ethical climate [33]. Furthermore, active help-seeking should be encouraged among the intensive care nurses against the related stigma [34]. The rare help-seeking behaviors can be attributed to the resilience and implicitness embedded in Chinese people’s characteristics [35]. Identifying the barriers in accessing supportive resources would inform the design of intervention to mitigate ethical conflict.

However, distinctive coping strategies were also shown in intensive care nurses. They displayed the strategy of engagement such as perspective-taking to deal with ethical conflict. Research indicated that nurses established greater therapeutic relationships with patients when they were able to adopt patient’s perspective [36]. This perspective-taking is a perceptual process that determines how an individual make meaning of the thoughts and feelings of another [37, 38]. It was positively associated with compassion satisfaction and played an essential role in the improvement of nurse-patient bond [39]. Consistent with prior study, our results proved the effectiveness of this coping strategy to produce vicarious feelings of patients and their family, which would be helpful to develop better understanding of their situations. However, it is interesting to note that being either empathetic or distressed can be influenced by the perspective of the observer. Intensive care nurses may become more mature and experienced when perceiving patients’ feelings than the new counterparts. This was supported by López-Pérez et al. who depicted that the accuracy of perspective-taking increased with the educational level and working experience of nurses [40]. This indicated the importance to enhance such empathic capacity and skills among newly-graduated nurses to cope with the ethical conflict in the ICU context.

Aside from perspective-taking, identifying the positive assets was also an engagement strategy in intensive care nurses, which was paid less attention in previous studies [10–13]. The factor behind this may lie in the non-confrontational personality traits in Chinese culture, which brought about positive reflection when dealing with ethical conflict [41]. We found that some of the intensive care nurses pondered the experience of ethical conflict when undergoing situational triggers. This would help them gain more insight into this issue and focus on the positive elements such as the motivation for learning, the responsibility of family, and the reflection on life instead of dwelling on the aftermath they cannot control. This strategy was beneficial for them to transform negative thoughts into optimistic beliefs and cultivate resilience, which was also reported in previous studies of grappling with work-related stress [42] and nursing care rationing [43].

It was also worth mentioning that ethical conflict was sensitive to culture and context. The impact of ethical conflict on intensive care nurses may vary from culture to culture and nation to nation. For example, the traditional western individualist usually gave priority to patients’ autonomy while the eastern collectivist tended to consider familial involvement central to navigating decision-making [44]. This would shape nurses’ views and coping strategies towards ethical conflict. Some defensive coping strategies such as detachment or avoidance in order to preserve the self are not uncommon, but generally not align with nurse’s primary obligation to the patient. The acceptance of self preservation can be explained by cultural differences. Culture incorporates various domains including language, religion, family traditions and social structure, and is dynamic, with cultural norms and practices evolving over time. Therefore, culture differences in the experience of coping with ethical conflict should be further explored, which would be helpful to discuss this topic across the borders.

This study was the first qualitative study that explored how intensive care nurses in China cope with ethical conflict in clinical practice, which is a novel aspect of our work. However, there are also several limitations in our study. First, all the participants came from a tertiary hospital located in a provincial capital city. Even though we used maximum variation sampling in the selection of research subjects to guarantee the representative, the sample from different cultural and economic backgrounds was not sufficiently represented. The second limitation was related to the methodology of the qualitative descriptive study, the result of which appeared not as robust as observational research. However, the amount of energy and time the researchers have spent on the process of data collection and analysis guaranteed the quality of the study.

### Implications

There are some implications for clinical practice and future research on ethical conflict. The results provide clues to help nurse administrators identify intensive care nurses’ vulnerability to ethical conflict and develop training and education programs to improve their moral agency and coping capacity. The identification of the existing coping strategies of intensive care nurses would assist in the better design and implementation of system-wide early counseling and supportive interventions. Academic educators can also get inspirations to prepare nurses to engage in these ethical conflicts in order to sustain their moral integrity [45]. Furthermore, this study lays the groundwork for future investigations. Instruments can be constructed to measure the coping behaviors of ethical conflict among intensive care nurses. The effectiveness of ethics education program need to be evaluated to inform the foundational curricula structures for the profession’s goal of preparing ethically competent nurses. Additionally, the mechanism of supportive interventions can be explored to mitigate the experience of ethical conflict and increase nurses’ engagement in ethical decisions. Lastly, this study would bridge the gap between health research and health policy and inform the decisions of policymakers and institutional stakeholders to optimize healthcare delivery system and guide system interventions.

## Conclusion

Intensive care nurses demonstrated differential experience of coping with ethical conflict in clinical nursing including problem-focused, emotion-focused and meaning-making strategies. However, these strategies were mainly on the individual level. Future research and clinical practice should explore supportive interventions and enhance the ethical environment on the system level. A better understanding of intensive care nurse’s coping experience would provide a basis for policymakers to optimize the policies and medical procedures, and for nursing administrators to deliver ethical education/training and institutional support for intensive care nurses to tackle this issue.

### Electronic supplementary material

Below is the link to the electronic supplementary material.

Supplementary Material 1

## Data Availability

The datasets used and/or analyzed during the current study are available from the corresponding author on reasonable request.
