# Supplementary material for: The experiences of intensive care nurses coping with ethical conflict: a qualitative descriptive study
Source: BMC Nurs. 2023 Nov 30;22:449. doi: 10.1186/s12912-023-01612-2 (PMC10687825; doi:10.1186/s12912-023-01612-2)
Supplement: Supplementary file 1 — Supplementary Material 1 [file 12912_2023_1612_MOESM1_ESM.docx]

**Supplementary table 1 Consolidated criteria for reporting qualitative studies (COREQ): 32-item checklist**

| No | | Item | Guide questions/description | Details |
| --- | --- | --- | --- | --- |
| Domain 1: Research team and reflexivity | | | | |
| Personal Characteristics | | | | |
| 1. | | Interviewer/facilitator | Which author/s conducted the interview or focus group? | The first author. |
| 2. | | Credentials | What were the researcher's credentials?*E.g. PhD, MD* | *PhD* |
| 3. | | Occupation | What was their occupation at the time of the study? | *PhD candidate.* |
| 4. | | Gender | Was the researcher male or female? | Female. |
| 5. | | Experience and training | What experience or training did the researcher have? | Have received qualitative study training. |
| Relationship with participants | | | | |
| 6. | | Relationship established | Was a relationship established prior to study commencement? | The interviewer had no contact with these participants before to guarantee the objectiveness of the results. |
| 7. | | Participant knowledge of the interviewer | What did the participants know about the researcher? e*.g. personal goals, reasons for doing the research* | The content of the research, the reasons for doing the research. |
| 8. | | Interviewer characteristics | What characteristics were reported about the interviewer/facilitator? e.g. *Bias, assumptions, reasons and interests in the research topic* | No bias. |
| Domain 2: study design | | | | |
| Theoretical framework | | | | |
| 9. | | Methodological orientation and Theory | What methodological orientation was stated to underpin the study? *e.g. grounded theory, discourse analysis, ethnography, phenomenology, content analysis* | Descriptive qualitative study. |
| Participant selection | | | | |
| 10. | | Sampling | How were participants selected? *e.g. purposive, convenience, consecutive, snowball* | Purposive sampling. |
| 11. | | Method of approach | How were participants approached? e*.g. face-to-face, telephone, mail, email* | Face-to face. |
| 12. | | Sample size | How many participants were in the study? | 15 intensive care nurse. |
| 13. | | Non-participation | How many people refused to participate or dropped out? Reasons? | None. |
| Setting | | | | |
| 14. | | Setting of data collection | Where was the data collected? e*.g. home, clinic, workplace* | Workplace. |
| 15. | | Presence of non-participants | Was anyone else present besides the participants and researchers? | No. |
| 16. | | Description of sample | What are the important characteristics of the sample? *e.g. demographic data, date* | In the demographic section. |
| Data collection | | | | |
| 17. | | Interview guide | Were questions, prompts, guides provided by the authors? Was it pilot tested? | Yes. |
| 18. | | Repeat interviews | Were repeat interviews carried out? If yes, how many? | No. |
| 19. | | Audio/visual recording | Did the research use audio or visual recording to collect the data? | Audio-recorded. |
| 20. | | Field notes | Were field notes made during and/or after the interview or focus group? | Field notes were made during the interviews. |
| 21. | | Duration | What was the duration of the interviews or focus group? | 50-73 min. |
| 22. | | Data saturation | Was data saturation discussed? | Yes. |
| 23. | | Transcripts returned | Were transcripts returned to participants for comment and/or correction? | Yes. |
| Domain 3: analysis and findings | | | | |
| Data analysis | | | | |
| 24. | Number of data coders | | How many data coders coded the data? | Two coders. |
| 25. | Description of the coding tree | | Did authors provide a description of the coding tree? | Yes. |
| 26. | Derivation of themes | | Were themes identified in advance or derived from the data? | Themes were derived from the data. |
| 27. | Software | | What software, if applicable, was used to manage the data? | Nvivo 12 (QSR International) |
| 28. | Participant checking | | Did participants provide feedback on the findings? | They approved the findings. |
| Reporting | | | | |
| 29. | | Quotations presented | Were participant quotations presented to illustrate the themes / findings? Was each quotation identified? e*.g. participant number* | Yes. |
| 30. | | Data and findings consistent | Was there consistency between the data presented and the findings? | Yes. |
| 31. | | Clarity of major themes | Were major themes clearly presented in the findings? | Two main themes were found. |
| 32. | | Clarity of minor themes | Is there a description of diverse cases or discussion of minor themes? | Four subthemes were showed. |
